# Contrasted impacts of commercial diets and rearing water on *Aedes aegypti* fitness and microbiota

**DOI:** 10.1128/msphere.00543-25

**Published:** 2025-11-10

**Authors:** Elodie Calvez, Isaure Quétel, Ludmina Saint-Alban, Gladys Gutiérrez-Bugallo, Christelle Dollin, Cédric Ramdini, Anubis Vega-Rúa

**Affiliations:** 1 Vector-Borne Diseases Laboratory, Environment and Health Research Department, Institut Pasteur de la Guadeloupe 27058, Les Abymes, Guadeloupe, France; 2 Microbial Ecology team, Environment and Health Research Department, Institut Pasteur de la Guadeloupe 27058, Les Abymes, Guadeloupe, France; 3 Department of Vector Control, Center for Research, Diagnostic, and Reference, Institute of Tropical Medicine Pedro Kourí, PAHO-WHO Collaborating Center for Dengue and its Control 549808, Havana, Cuba; 4 Vector Control Service of Grande-Terre, Regional Health Agency, Airport Zone South Raizet, Les Abymes, Guadeloupe, France; University of Michigan Medical School, Ann Arbor, Michigan, USA

**Keywords:** *Aedes aegypti*, diet, larval site, fitness, microbiota

## Abstract

Mosquito rearing optimization in laboratory conditions is crucial for both vector research and control. Although the addition of nutrients is important for *Aedes aegypti* development from immature stages to adult mosquitoes, little is known about the nutrient composition of commercial diets used for mosquito rearing and their influence on *Ae. aegypti* life traits. Here, we evaluated the influence of four commercial diets commonly used to rear *Ae. aegypti* in the laboratory on its fitness, lifespan, and microbiota. We also compared the effect of these diets on this mosquito when combined with two different rearing waters (laboratory versus field-collected waters). Our investigations demonstrated that higher levels of protein and lipid in commercial diets promote better *Ae. aegypti* development, lifespan, and size in both water. Metagenomic analysis revealed specific modulations of adult microbiota composition according to both diet and rearing water. *Chryseobacterium* dominated the microbiota of female mosquitoes reared in laboratory water, except for yeast condition, where a more diverse microbiota was observed. When reared in larval site water, the microbiota diversity was overall higher despite diet addition, except for fish food, which promoted *Sphingobacterium* dominance. Given the pivotal influence of diet addition during the larval stage on *Ae. aegypti* microbiota and life traits, rearing conditions should be carefully chosen according to the goals of the research (i.e., vectorial capacity estimations) or vector control intervention.

**IMPORTANCE:**

*Aedes aegypti* is the main vector of arbovirus, such as dengue, yellow fever, and chikungunya viruses. Vector research and control are primarily carried out in laboratories, with larval stage rearing conducted using commercial diet. If many nutrients are essential for *Ae. aegypti* development, gaining insight into the influence of these diets and their nutrient levels is important to promote optimized rearing worldwide. In this study, our results indicated a significant impact of commercial diet on *Ae. aegypti* development, lifespan, size, and microbiota related to contrasted protein, lipid, and carbohydrate levels in these diets. This study will help people working with *Ae. aegypti* raise awareness in staff working with *Ae. aegypti* to select optimized diets for their specific purpose.

## INTRODUCTION

*Aedes aegypti* is the main vector of arboviruses, such as dengue, yellow fever, Zika, and chikungunya viruses, in tropical and subtropical regions (1–3). Immature stages of this mosquito develop in larval sites whose abiotic and biotic factors, including temperature, microbiota, and nutrient content, influence their development and have been shown to translate into morphological and physiological modifications in the emerging adults (4–6). Understanding the effects of these factors on *Ae. aegypti* is recommended to choose appropriate rearing conditions for this species in the laboratory.

Optimization of mosquito immature larvae rearing is important for (i) entomological surveillance (i.e., identification of adults that were field-collected as larvae), (ii) research purposes, such as dissecting pathogen transmission abilities in mosquitoes or evaluating their levels of insecticide resistance, and (iii) the implementation of vector control approaches requiring mosquito mass-rearing (i.e., sterile insect technique, *Wolbachia-*based population introgression strategy (7). Mosquito rearing is conducted, for the immature stages, in water containing a given microbial composition and supplemented with diet. Essential nutrients for mosquito development and growth are classified in two groups: macronutrients (carbohydrates, amino acids, and polyunsaturated fatty acids) and micronutrients (vitamins, salt, sterols, and metals) (6, 8–11). The addition of diet and, therefore, of specific nutrients during the larval stage has been shown to influence several mosquito traits, such as larval development, adult longevity, ability to fly, or body size (11, 12). Additionally, bacteria present in the larval site water could also influence *Ae. aegypti* microbiota (13–15). Moreover, the presence of microorganisms is also an important factor for nutrient assimilation and influences mosquito development (13, 16–19).

Many options are available for larval feeding in the laboratory. One of the most common is the use of commercial diets, such as yeast or animal feed (i.e. ,TetraMin flakes, rabbit pellets) (9, 20, 21). Although the influence of nutrient content on vector development has been previously investigated (15, 22), little is known about the nutrient composition of the commercial diets and their influence on *Ae. aegypti* life traits and microbiota (12, 21). Here, we assess how commercial diets with different macronutrient content and microbiota impacted *Ae. aegypti* and provide a holistic overview of *Ae. aegypti* phenotypic differences associated with diets commonly used in laboratory. To achieve this goal, we characterized the influence of four commercial diets added into waters with different microbial compositions (laboratory and field collected waters) on *Ae. aegypti* fitness, lifespan, and microbiota, all these being important factors influencing the mosquito vectorial capacity. Beyond the contribution to fundamental knowledge on microbiota-macronutrients-mosquito interaction, we believe the data obtained in this study will raise awareness in staff working with *Ae. aegypti* to select optimized diets for their specific purpose.

## RESULTS

### Rabbit food, lacking in proteins and lipids, delayed *Ae. aegypti* development compared to the other commercial diets

**Fig 1:**
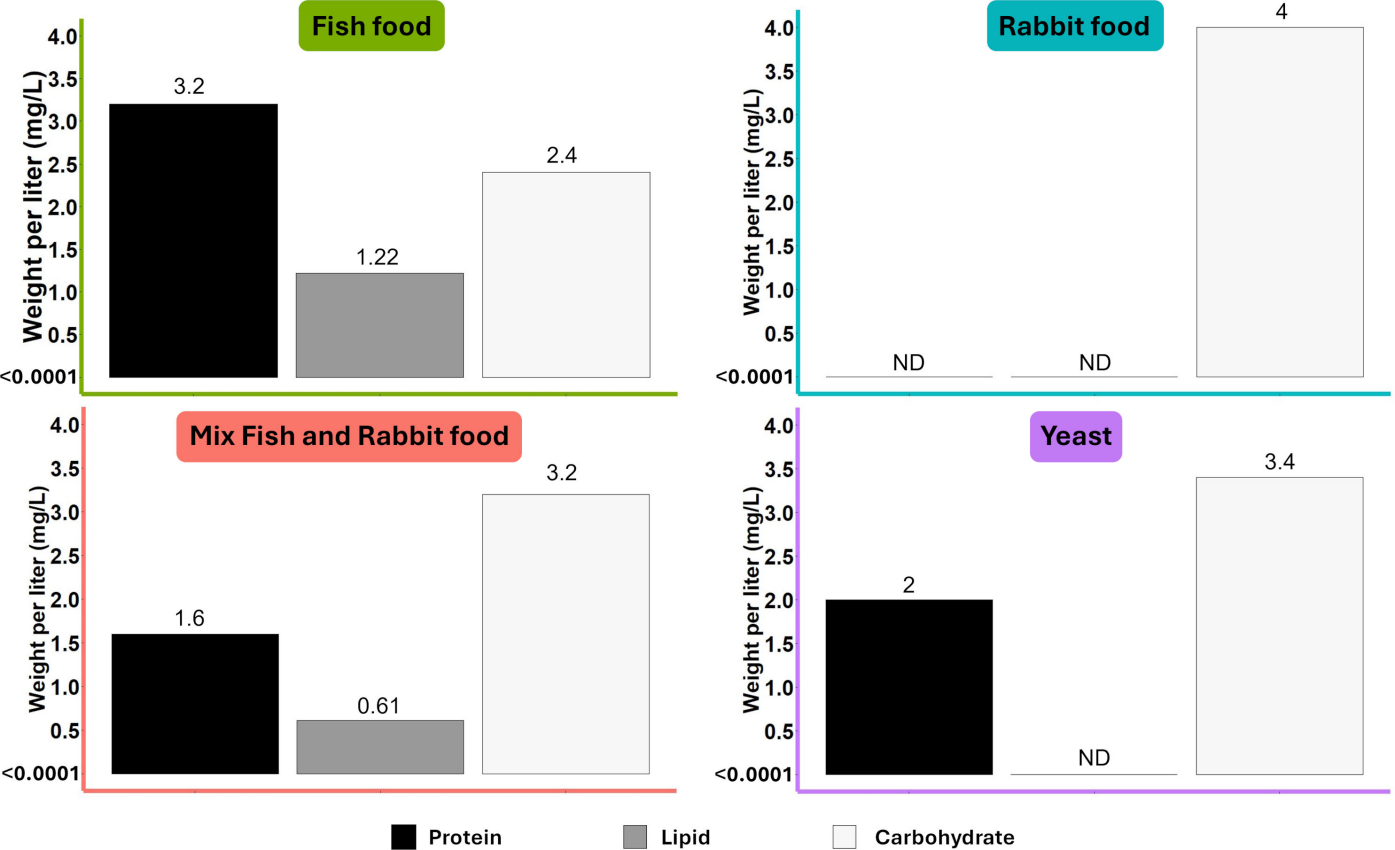
Nutritional composition in the water after diet addition. Concentration expressed in mg/L according to the parameters applied in this study: 0.2 g of diet diluted in 1 L of water.

**Fig 2:**
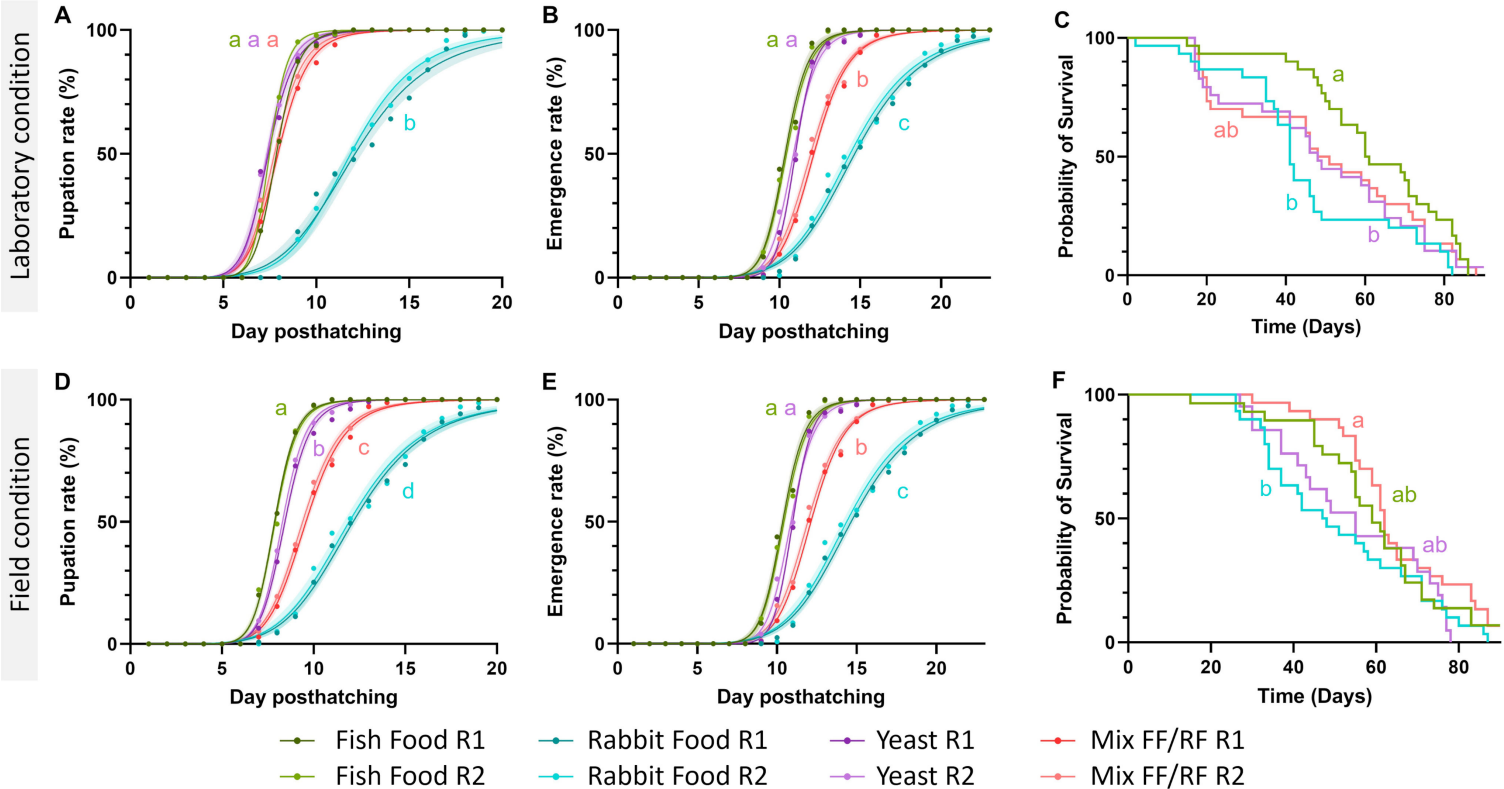
*Aedes aegypti* development and survival depending on the rearing diet and water origin (laboratory versus field-collected water). Pupation rate, emergence rates (both *N* = 250 and 2 replicates per condition), and female survival according to the four diets used were estimated during rearing either with dechlorinated laboratory tap water (**A, B, and C**, respectively) or with water collected from breeding sites in the field (**D, E, and F**, respectively). The lines represent nonlinear regression obtained for each diet and replicate; the color-shaded areas represent 95% confidence intervals. For survival analysis (**C and F**), *Ae. aegypti* Kaplan-Meier curves are displayed. Different superscript letters indicate significant differences (*P* < 0.05) between rearing diet for a given water type.

The four commercial diets used in the experiments were fish food flakes (FF), yeast, rabbit food pellets (RF), and a mix 1:1 of fish and rabbit food (Mix FF/RF). These diets contain different macronutrient levels (Fig. 1). In our experimental condition (0.2 g of diet in 1 L of water), macronutrient dosage indicated the presence of carbohydrate in all the diets selected (>2.4 mg/L). The presence of protein and lipid was also detected in FF and mix FF/RF (between 0.61 and 3.2 mg/L), whereas lipid appeared absent in yeast (<0.0001 mg/L). In RF, protein and lipid were both undetectable (<0.0001 mg/L). To assess the difference in *Ae. aegypti* development according to these diets used for rearing the immature stages, we compared the time needed for 50% of larvae to pupate and for 50% of pupae to emerge as adults. For rearing conditions using laboratory water, between 7.36 and 7.94 days were needed to obtain 50% pupation with FF, yeast, and the mix FF/RF diets, while 11.97 days on average were needed for RF, highlighting a significant development delay for this latter diet (*P* < 0.0001, analysis of variance [ANOVA], Tukey test) (Fig. 2A). Regarding the time required for 50% of emergence rate, homogeneous results were found for FF and yeast (mean of 10.66 days) (Fig. 2B), both being significantly shorter than for the mix FF/RF (mean of 12.03 days; respectively *P* = 0.0008 and *P* = 0.0038, ANOVA, Tukey test) and for RF (mean of 14.51 days) that exhibit the longest emergence time of all diets (*P* < 0.0001 compared to FF and yeast, *P* = 0.0002 compared to mix FF/RF, ANOVA, Tukey test).

In field-collected water, the time needed for 50% pupation also differed between diets (Fig. 2D). The longest time was recorded for RF, as previously observed with laboratory water (mean = 12.05 days; *P* < 0.0001, ANOVA, Tukey test). Significant differences were also found between the mix FF/RF (mean = 9.49 days) and the two other diets (mean = 8.37 days for yeast [*P* = 0.0018] and mean = 7.89 days for FF [*P* = 0.0004], ANOVA, Tukey test). Time for 50% pupation also significantly differed between yeast and FF (*P* = 0.0395, ANOVA, Tukey test). Regarding emergence rates (Fig. 2E), the time needed for 50% emergence was similar for yeast and FF (mean of 10.66) but significantly shorter compared to RF (mean of 14.51 days; *P* < 0.0001, ANOVA, Tukey test) and the mix FF/RF (mean = 12.03 days; *P* = 0.0008 compared to FF, *P* = 0.0038 compared to yeast and *P* = 0.0002 compared to RF, ANOVA, Tukey test).

Interestingly, time for 50% pupation was significantly shorter in laboratory water compared to field-collected water for yeast and mix FF/RF diets. For yeast, 50% pupation was delayed in field-collected water (8.37 days) compared to laboratory water (7.39 days) (*P* = 0.0019, ANOVA, Tukey test). For mix FF/RF, an increase of the mean time needed for 50% pupation was recorded from 7.85 to 9.49 days for laboratory and field-collected waters, respectively (*P* < 0.0001, ANOVA, Tukey test). No significant differences were recorded for the other diets or at the emergence stage.

### Higher *Ae. aegypti* female survival with diets containing a higher amount of protein and lipid independently of water origin

To evaluate the influence of larval diet and the water used for rearing on female *Ae. aegypti* lifespan, we recorded daily the number of dead adult mosquitoes and estimated survival for each of the eight rearing conditions. In laboratory water, no significant differences were found between the FF diet and the mix FF/RF (Fig. 2C). However, lifespan was significantly shorter with RF and yeast compared to the condition with FF (RF: *P* = 0.0003 and yeast: *P* = 0.025, Gehan-Breslow-Wilcoxon test). In field-collected water, lifespan was more homogeneous with a shorter survival only recorded for RF compared to the mix FF/RF (*P* = 0.0053, Gehan-Breslow-Wilcoxon test) (Fig. 2F). Water origin did not influence *Ae. aegypti* survival when reared with FF, yeast, and RF. However, for the mix FF/RF, a longer lifespan was observed after rearing in field-collected water compared to laboratory water (*P* = 0.0175, Gehan-Breslow-Wilcoxon test).

### Fish food and yeast diets led to *Ae. aegypti* female with longer wing length in both laboratory and field-collected waters

**Fig 3:**
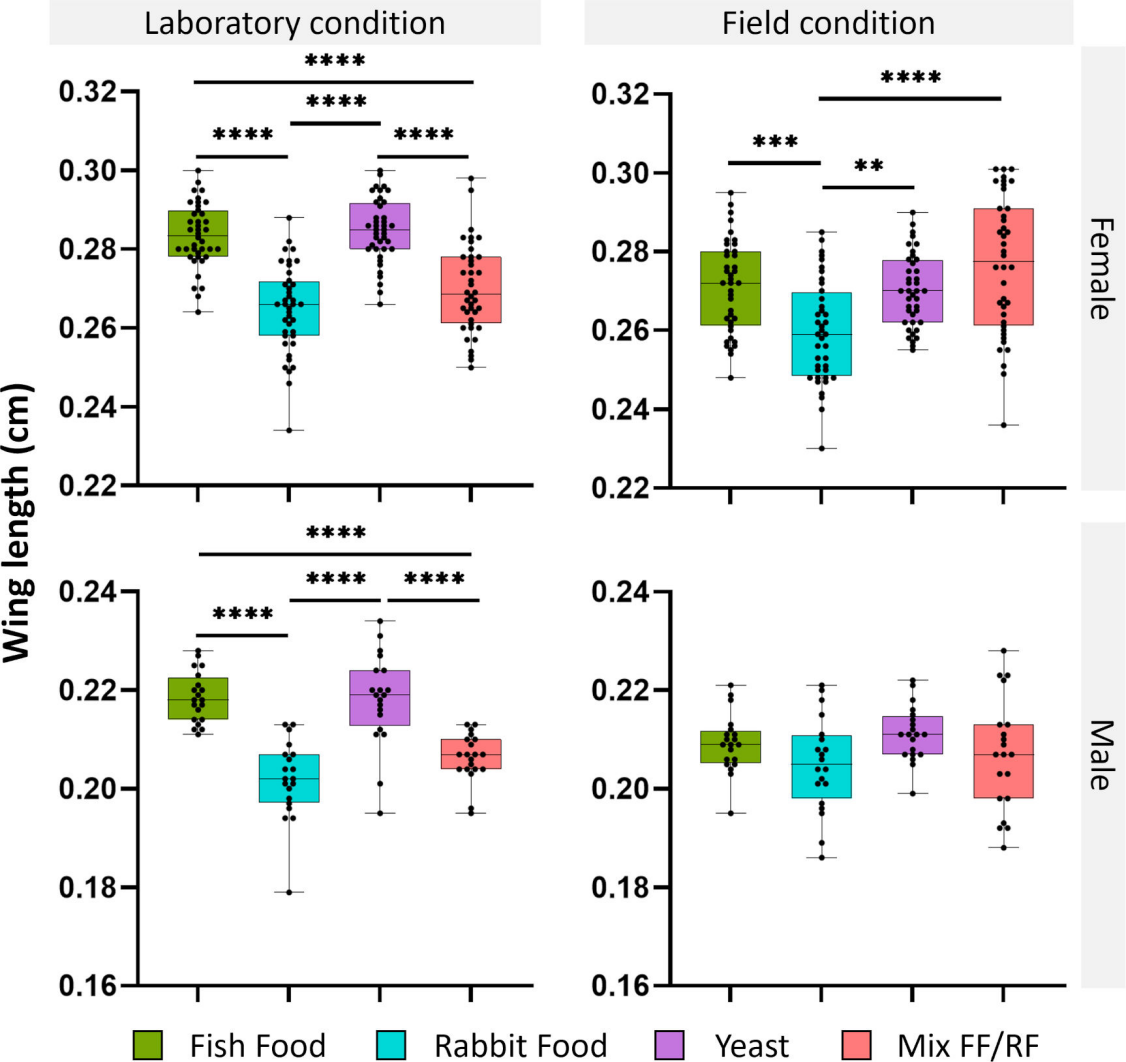
*Aedes aegypti* wing length from male and female mosquitoes reared at larval stage under different dietary and water conditions (laboratory versus field-collected water). The wing length was measured for newly emerged adult females (*N* = 40) and males (*N* = 20) whose larvae were reared either with dechlorinated laboratory tap water or water collected from breeding sites in the field independently supplemented with four different commercial diets. Significant differences are indicated by asterisks (**P < 0.01; ***P < 0.001; and ****P < 0.0001). The bars indicate the minimum and maximum values for each condition.

To investigate the influence of both commercial diets and water origin on *Ae. aegypti* body size, we measured the wing length (from the tip to the distal end of the allula) of females and males. In both *Ae. aegypti* females and males reared in laboratory water (Fig. 3), wings of mosquitoes fed with FF and yeast (mean length for females 0.28 cm and for males 0.22 cm for both) were significantly longer than those of mosquitoes fed with RF (mean length for females 0.26 cm and for males 0.20 cm) and the mix FF/RF (mean length for females 0.27 cm and for males 0.21 cm) (*P* < 0.0001, ANOVA, Tukey test). Regarding mosquitoes reared in field-collected water, wings of females fed with RF (mean length 0.26 cm) were significantly smaller compared to those fed on FF (length 0.27 cm; *P* = 0.0006, ANOVA, Tukey test), yeast (length 0.27 cm; *P* = 0.0027, ANOVA, Tukey test), and the mix FF/RF (length 0.28 cm; *P* < 0.0001, ANOVA, Tukey test) (Fig. 3). Interestingly, no significant differences were found between the four diets for males (mean length 0.21 cm; ranging from 0.21 cm for yeast to 0.20 cm for RR).

Significant differences were also found for some conditions between the rearing in laboratory and field-collected waters. Indeed, both females and males fed with FF had significantly longer wing length after rearing in laboratory water (*P* < 0.0001 and *P* = 0.0057, respectively, ANOVA, Tukey test). Similar results were also recorded for females fed with yeast, with longer wing length observed for females reared in laboratory water compared to those reared in field-collected water (*P* < 0.0001, ANOVA, Tukey test).

### Emerging mosquitoes harbor a more diverse microbiota composition when their larvae are reared with yeast in laboratory water

**Fig 4:**
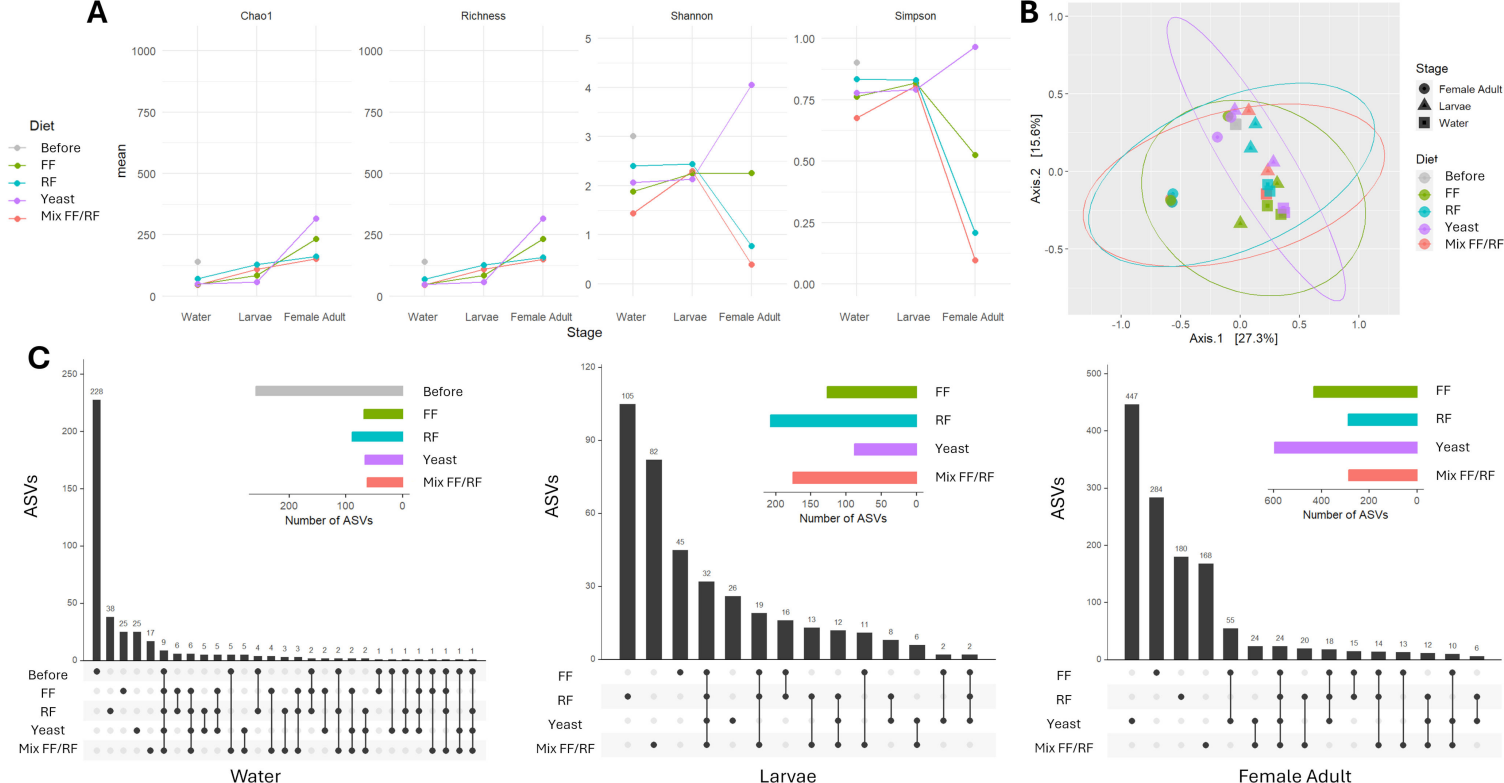
Influence of diet addition for larval rearing in dechlorinated tap water on microbiota diversity in *Aedes aegypti*. (**A**) Alpha indices, (**B**) beta diversity, and (**C**) upset plots displaying ASV abundance and overlap between rearing conditions for the three types of samples analyzed: breeding site water, larvae (two pools of 10 fourth instar larvae), and adult females (two pools of 10 newly emerged adults).

To determine the influence of diet addition on *Ae. aegypti* microbiota in laboratory water, 16S rRNA gene metabarcoding was separately performed on water, larvae, and newly emerged females reared with the different diets in dechlorinated tap water. In total, 1,860 amplicon sequencing variants (ASVs) were identified from the samples tested (water, larvae, and adult females) belonging to 16 phyla and 210 genera. Whatever the diet used, bacterial communities’ richness (alpha diversity) decreased after diet addition (from mean 141.5 before to lower than 76 after diet addition) but remained stable between the water and the larvae (mean richness 74.6, min = 34, max = 133; Fig. 4A). Interestingly, an increase in the richness was observed at the adult stage ranging from 153 (mix FF/RF) to 316.5 (yeast). However, alpha diversity based on Shannon and Simpson indices decreased for all the conditions, except for yeast, between larvae and adult mosquitoes (Fig. 4A).

The variation of microbiota diversity (alpha diversity) in each sample demonstrated a significant difference at the water level between the diets tested (*P* = 0.0024, Shannon and *P* = 0.0099, Simpson, ANOVA) (Fig. 4A). Alpha diversity was significantly higher for condition “Before diet addition” compared to FF (*P* = 0.008, Shannon, ANOVA, Tukey test), yeast (*P* = 0.0175, Shannon, ANOVA, Tukey test), and mix FF/RF (*P* = 0.0018, Shannon and *P* = 0.0072, Simpson, ANOVA, Tukey test). Furthermore, alpha diversity was also significantly higher for RF compared to mix FF/RF (*P* = 0.0153, Shannon and *P* = 0.0318, Simpson, ANOVA, Tukey test). Difference between the diet among the microbial composition (beta diversity) was observed (*R*²= 0.1981, *P* = 0.01, permutational multivariate analysis of variance [PERMANOVA] statistical test) as in water (*R*²= 0.7168, *P* = 0.004, PERMANOVA statistical test) (Fig. 4B).

**Fig 5:**
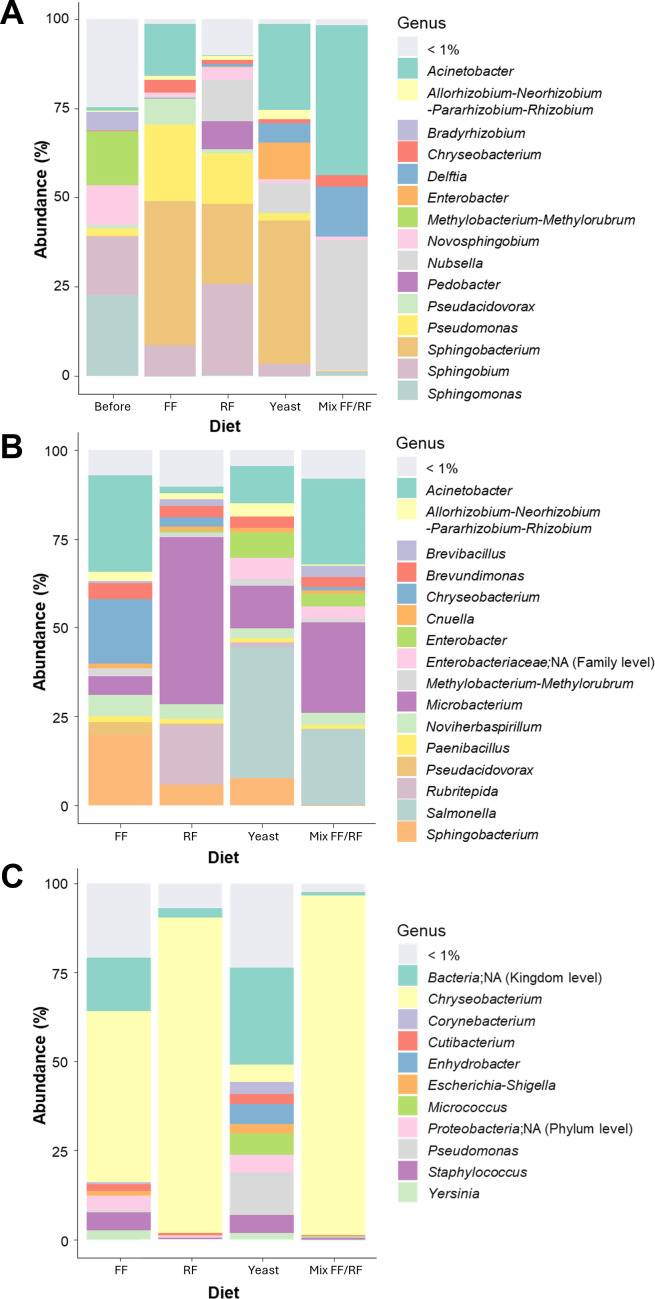
Impact of diet addition for larval rearing in dechlorinated tap water on *Aedes aegypti* microbiota composition. Investigations were conducted in (**A**) breeding site water, (**B**) larvae (fourth instar larvae; two pools of 10 individuals), and (**C**) emerging adult females (two pools of 10 adults) to determine the percentage of the major bacterial genera (>1%).

Interestingly, a high amount of specific ASVs (*N* = 228) was found before the addition of any diet in the water (Fig. 4C). Microbiota analysis of laboratory water demonstrated a predominance of minor genera with less than 1% of relative abundance (24.70%) (Fig. 5A). After the addition of the diet, the numbers of ASVs were lower (<70.5), and the most abundant ASVs appeared to be specific to each condition (between 17 for mx FF/RF and 38 for RF) (Fig. 4C). Indeed, the addition of FF or yeast enriched microbiota with *Sphingobacterium* (~40%) was mainly associated with *Pseudomonas* (21.40%) for FF and *Acinetobacter* (23.91%) for yeast (Fig. 5A). For the RF condition, *Sphingobium* (25.36%) and *Sphingobacterium* (22.62%) were the most abundant genera, whereas in mix FF/RF, there were *Acinetobacter* (41.97%) and *Nubsella* (36.59%) (Fig. 5A). The presence of specific ASVs was also recorded in mosquitoes at larval and adult stages (Fig. 4C). At the larval stage, the microbiota composition differed between the four diets used (Fig. 5B). Indeed, larvae rearing with RF led to a high abundance of *Microbacterium* (46.83%). *Microbacterium* (25.27%), *Acinetobacter* (23.94%), and *Salmonella* (21.59%) were predominantly found in larvae reared with mix FF/RF (Fig. 5B). Larvae reared with yeast presented a predominance of *Salmonella* (36.95%), while for the FF condition, *Acinetobacter* (27.33%), *Sphingobacterium* (19.73%), and *Chryseobacterium* (18.20%) were mainly detected. At the adult stage, the microbiota of *Ae. aegypti* females reared in water supplemented with FF, RF, and mix FF/RF was mainly composed of *Chryseobacterium* at contrasted proportions (47.87, 88.57, and 95.07%, respectively) (Fig. 5C). The microbiota composition diversity of female previously reared in the presence of yeast was higher compared to the other conditions with a predominance of unidentified bacteria (27.30%) and minor genera (23.74%), followed by 10 other bacteria with similar relative abundances (ranging from 1.80 to 11.93%) (Fig. 5C).

### Diets containing lipids promote the presence of specific predominant genera in *Ae. aegypti* microbiota at the adult stage after rearing in field-collected water

**Fig 6:**
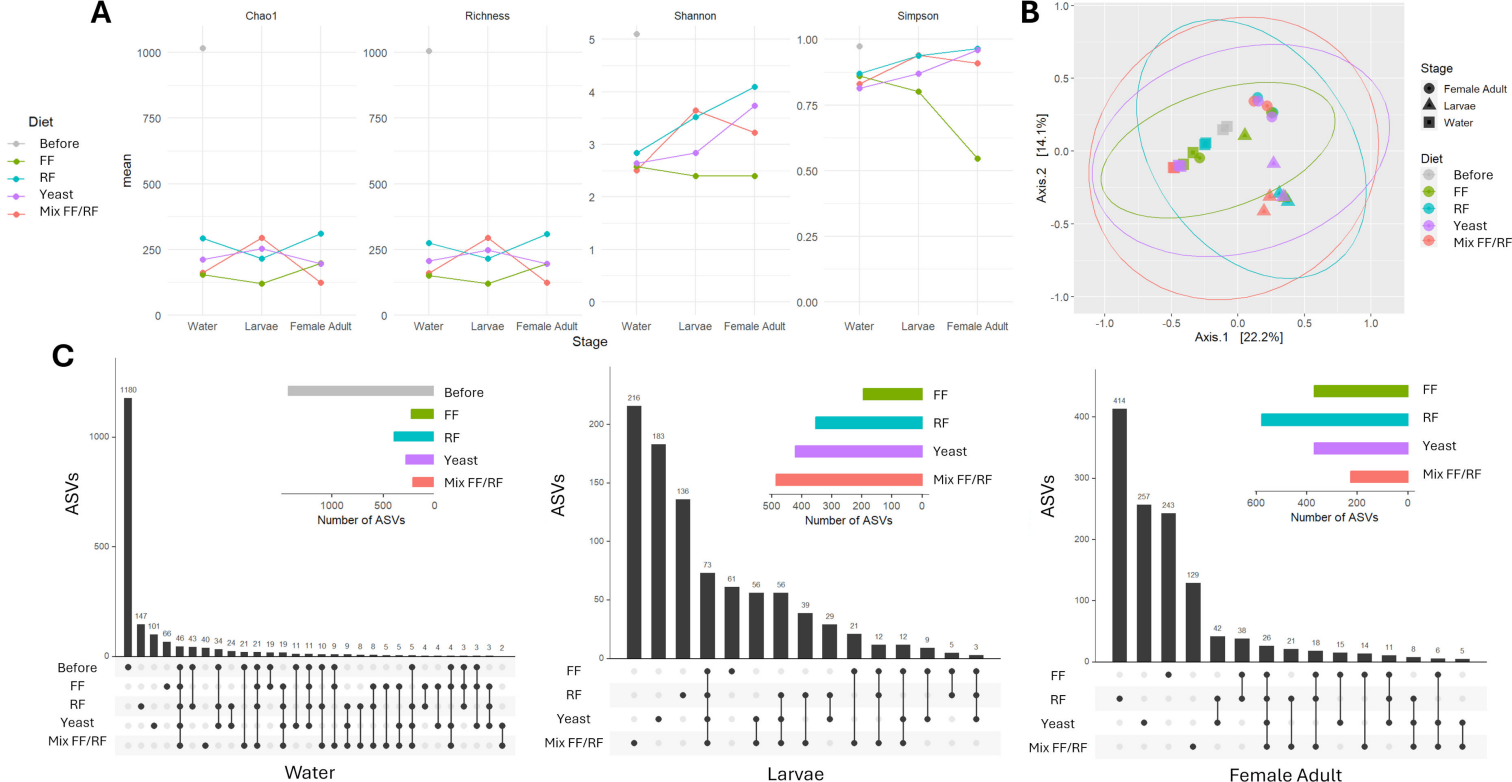
Influence of larval diet addition in field-collected water on *Aedes aegypti* microbiota diversity. (**A**) Alpha and (**B**) beta diversity indices and (**C**) upset plots according to each condition and for the three stages analyzed: water, larvae (fourth instar larvae; two pools of 10), and female adult (two pools of 10 newly emerged adults).

We also investigated how diet addition influenced the microbiota of field-collected water, as well as that of larvae and newly emerged females reared under these conditions. In field-collected water, a total of 3,679 ASVs was detected among the samples tested (water, larvae, and newly emerged females) belonging to more than 40 phyla and 462 genera. Analysis of the mean richness revealed a dramatic decrease after diet addition in the water from mean 1,017 to <331 (Fig. 6A). The richness then remained stable between water, larvae, and female adults (between 89 and 426) (Fig. 6A).

Significant differences in microbiota diversity were only found in water samples (*P* < 0.0001, Shannon; *P* = 0.0087, observed; *P* = 0.0087, Chao1, and *P* = 0.0011, Simpson, ANOVA), especially before and after diet addition (*P* < 0.0001, Shannon; *P* < 0.0242, observed; *P* < 0.0243, Chao1 and *P* < 0.006; Simpson ANOVA, Tukey test) (Fig. 6A). Microbiota composition was significantly different (*R*²= 0.1994, *P* = 0.013, PERMANOVA statistical test) as in the water according to the diet status (*R*²= 0.7846, *P* = 0.002, PERMANOVA statistical test) (Fig. 6B).

**Fig 7:**
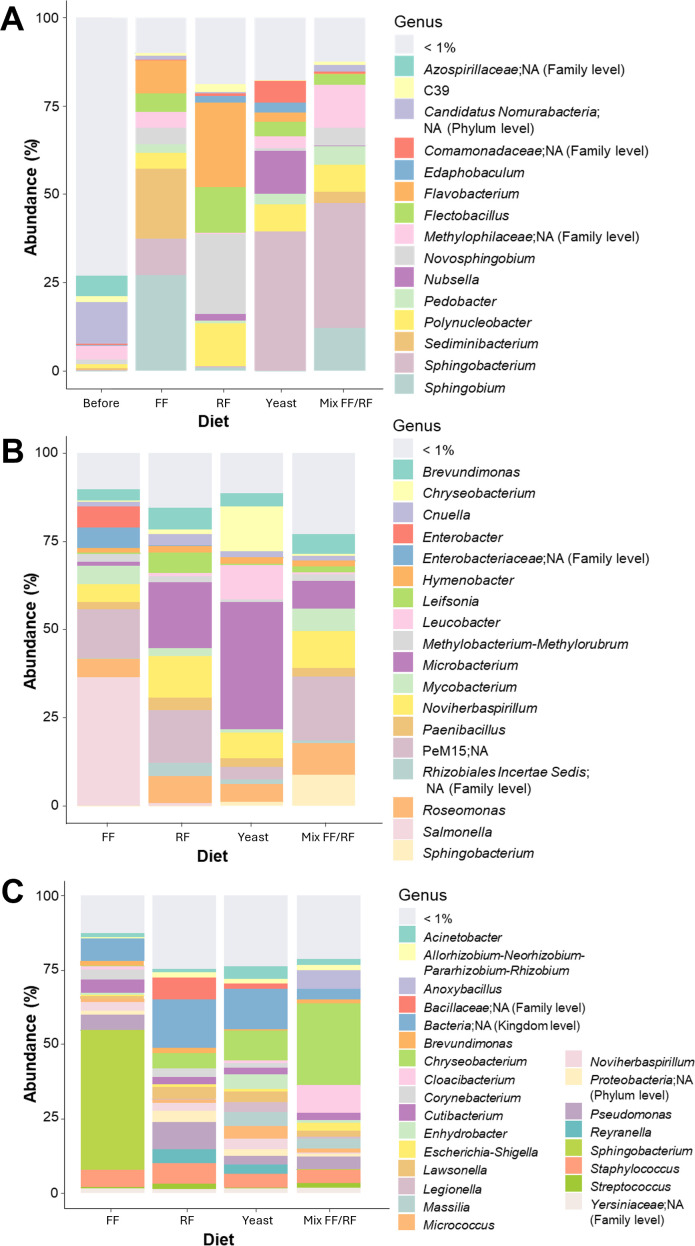
Impact of larval diet addition in field-collected water on the dominant bacterial genera present in *Aedes aegypti* microbiota. Investigations were conducted in (**A**) breeding site water, (**B**) larvae (fourth instar: two pools of 10), and (**C**) female adults (two pools of 10 newly emerged adults) to determine the percentage of the major bacterial genera (>1%).

As for laboratory water, most abundant ASVs were mostly specific to each condition in water at the larval and female adult stages (Fig. 6C). Analysis of microbiota composition demonstrated a high proportion of minor genera (73%) in the field-collected water before the addition of a diet (Fig. 7A). After the addition of yeast or mix FF/RF, *Sphingobacterium* was detected as the major genera (>35.32%). In FF condition, *Sphingobium* and *Sediminibacterium* were the most abundant genera (27.08 and 19.78%, respectively), whereas in RF condition, it was *Novosphingobium* and *Flavobacterium* (22.75 and 24.09%, respectively). At the larval stage, the most abundant genera were *Salmonella* for FF condition (36.34%) and *Microbacterium* for yeast (36.06%), while genus diversity was high for RF and mix FF/RF conditions (Fig. 7B). After larval rearing in field-collected water, newly emerged female adults presented a high genus diversity in the microbiota composition for the RF and yeast conditions. For FF and mix FF/RF conditions, even if the genera diversity was also high, *Sphingobacterium* and *Chryseobacterium* represented 47.07 and 27.20% of relative abundance, respectively (Fig. 7C).

### Differential impact of diet addition after larval rearing in laboratory or field-collected water on *Ae. aegypti* microbiota

Overall, the mean ASV richness was significantly higher in field-collected water compared to laboratory water both before and after diet addition (*P* = 0.00589, ANOVA) (Fig. 4A and 6A). The same observation was done at the larvae stage, except for FF with a homogenous richness for both water conditions (laboratory mean = 84.5; field-collected mean = 121). At the adult stage, the richness appeared to be dependent on both water and diet combinations. In both waters, ASVs appeared to be specific to the conditions studied in the water and at the larval and adult stages (Fig. 4C and 6C). After diet addition, microbiota composition in water was specific to FF, RF, and mix FF/RF conditions but not for yeast that showed a common predominance of *Sphingobacterium* whatever the water used (>38.58%) (Fig. 5A and 7A). At the larval stage, with diverse proportions, *Microbacterium* seemed to be an abundant genus in both waters for yeast, RF, and mix FF/RF (abundance between 7.97% for mix FF/RF in field-collected water and 46.83% for RF in laboratory water) (Fig. 5B and 7B). At the adult stage (Fig. 5C and 7C), the microbiota composition of female *Ae. aegypti* seemed diverse in both waters for yeast, whereas *Chryseobacterium* appeared to be predominant in adults after a rearing with mix FF/RF in both laboratory and field-collected water (95.07 and 27.20%, respectively). For RF, adult microbiota composition was mainly composed of *Chryseobacterium* (88.57%) after rearing in laboratory water and highly diverse after rearing in field-collected water. For FF, *Chryseobacterium* was the predominant genus in adult microbiota after a rearing in laboratory water (47.87%), whereas it was *Sphingobacterium* after a rearing in field-collected water (47.07%). Overall, these results demonstrated the importance of both water origin and diet in the specific microbiota establishment in *Ae. aegypti*.

## DISCUSSION

For decades, mosquito rearing has been performed in laboratories using commercial diets for research or vector control strategies (7). If special attention has been given to homemade diets and nutrients for mosquito rearing optimization, little is known about the influence of commercial diets used at the larval stage and their macronutrient content on *Ae. aegypti* life traits and microbiota. This lack of knowledge could be explained by the diversity of rearing methods used and the complexity of interactions between the environmental and rearing parameters selected (e.g., temperature, diet, larval density) (23). In this study, we investigated under controlled and standardized conditions the impact of four commercial diets using two rearing waters (laboratory tap water or field-collected water) on *Ae. aegypti* development, size, lifespan, and microbiota.

First, we evaluated the influence of these diets with contrasted concentrations of macronutrient content (lipids, proteins, and carbohydrates) on *Ae. aegypti* development and survival after rearing them in laboratory water. Furthermore, we evaluated these aspects using field-collected water, as *Ae. aegypti* larval sites could also influence its development and microbiota (13). Heterogeneous levels of nutrients, physicochemical properties, and a high level of diversity in microbiota composition have also been observed in larval sites in the field (13, 14, 24, 25). In this study, we evaluated whether microbiota differences in the water used for rearing (laboratory vs field-collected waters) could modify the outcome of diet addition on *Ae. aegypti* life traits. The contrasted nutrient content of our diets allowed us to conclude that when larvae were fed with an RF diet that contains a low amount of protein and lipid concentrations, the development of *Ae. aegypti* was delayed compared to other diets in both water conditions. However, a lack of lipids can be counterbalanced by carbohydrates as observed with yeast. Previous studies demonstrated that a high carbohydrate amount is required to allow an optimized (quick and homogenous) rearing, especially for the larval growth, when the threshold concentration of lipid and protein is too low (6, 9, 26–28). However, an excess of protein could be harmful for *Ae. aegypti* development probably due to a toxic production of ammonia during protein digestion (6). Additionally, larvae fed with FF and mix FF/RF demonstrated a longer lifespan compared to larvae fed with other diets, especially in laboratory water, demonstrating the importance of larval diet on *Ae. aegypti* survival (29, 30). The presence of carbohydrates appeared to be an important factor for *Ae. aegypti* growth, especially when protein concentration is low. Lipids can be produced from carbohydrate resources, which could explain the complete development of the mosquitoes in the RF condition in both waters (6, 26). Furthermore, wing analysis revealed that both male and female *Ae. aegypti* reared with FF and yeast had longer wing length compared to the specimens reared with other diets in laboratory water. In field water, smaller females were obtained with RF compared to other diets, while no impact was observed in male mosquitoes’ wing length. Diet seemed to impact more drastically *Ae. aegypti* female size as previously demonstrated by Van Schoor et al. (6).

The second aim of this study was to evaluate how the larval diet influenced *Ae. aegypti* microbiota during a standardized rearing procedure. The influence of the larval site on *Ae. aegypti* microbiota was previously highlighted in field and laboratory water (13, 14, 31–33). Our results demonstrated both in laboratory and field-collected waters a decrease of ASV richness after diet addition. Water microbiota diversity and composition were also significantly modified by the diet and influenced *Ae. aegypti* microbiota composition during its development until adulthood. In our study, we found a predominance of different bacteria, such as *Sphingobacterium*, after addition of FF, RF, and yeast in laboratory water, which suggests a diet-associated selection of bacteria. Hence, the genera best adapted to the nutrients added by the diet will more likely become predominant. Protein, carbohydrate, and lipid are essential for bacteria growth and used, for example, as a source of carbon (34). In our study, nutrient quantification was performed on the total lipid, protein, or carbohydrate content. It could be interesting in further experiments to determine if the presence of specific nutrients (e.g., glucose, fructose, or maltose for carbohydrate) enhances the presence of predominant genera by the activation of specific bacterial metabolic pathways (e.g., glycolysis) (35). Interestingly, this diet-associated impact on microbiota is less observable in adult mosquitoes. Our experiments conducted with laboratory water showed that females harbored a similar microbiota characterized by a *Chryseobacterium* predominance whatever the diet used, except for yeast. When fed with yeast, females showed a highly diverse microbiota composed mainly of an unidentified bacteria and minor genera. Conversely, when reared in field-collected water, female microbiota composition was highly diverse, except when nourished with FF, where *Chryseobacterium* predominated, as previously observed in experiments with laboratory water*.* This genus is commonly found in mosquito microbiota, especially in newly emerged *Ae. aegypti*, as in our study (17, 36). Overall, despite the diet status, microbiota composition was more diverse after larval rearing in field-collected water. Given that microbiota composition and diversity could influence mosquito immunity, fitness, blood digestion, or virus transmission (37–42), further investigations are needed to evaluate the impact of the nutrients used for immature stages rearing on *Ae. aegypti* ability to transmit arboviruses. This is particularly important when alternative vector control strategies based on mosquito mass-rearing are increasingly being implemented (43). In view of our findings, it could be interesting to investigate further *Chryseobacterium*-arboviruses interactions. Previously, it was found that the abundance of this genus was higher in *Ae. aegypti* mosquitoes fed with ZIKV-spiked blood compared to the noninfectious blood and sucrose/water groups (44).

Taken together, our data demonstrated how diets commonly used in laboratories impacted *Ae. aegypti* development, lifespan, and microbiota. Our findings demonstrated that protein content in commercial diets (i.e., FF, yeast, and mix FF/RF) is pivotal to ensure a quick and homogenous *Ae. aegypti* rearing (pupation and emergence) and a good mosquito survival. Conversely, when the commercial diet did not contain lipids and proteins (i.e., RF), the development was delayed. Secondly, FF and yeast provided mosquitoes with longer wing length for both females and males. Finally, the different diets influenced the establishment of *Ae. aegypti* microbiota through important modifications of rearing water microbiota. However, yeast yielded adults harboring a more diverse microbiota composition closer to those that can be found in the field (14). Based on our results (Table 1), for example, to incriminate a local mosquito as a vector for a given pathogen and assess the local epidemic risk, yeast diet can be a good choice because it allows a very good mosquito development and yields adult mosquitoes with a more diverse microbiota like the ones observed in the field. However, if the goal is to conduct a mosquito mass rearing and to have a quick larval development and adult mosquitoes of bigger size (i.e., to increase flight ability and dispersal), diets with at least protein and carbohydrate like yeast, fish food, and mix FF/RF could be more adapted. Finally, adding diet to field-collected water could be interesting in certain contexts given that the adult mosquitoes could develop well, and their microbiota could be diverse as found in the field (17).

**TABLE 1:** Summary of the effect observed in this study in both waters^*a*^ according to the different diet used compared to the fish food diet (used as reference^*b*^)

Effect	FF^*f*^	Yeast	Mix FF/RF	RF^*g*^
Delay for pupation^*c*^	NA^*e*^	No	No	Yes
Delay for emergence^*c*^	NA	No	No	Yes
Decrease of survival	NA	Laboratory water: yes Field-collected water: no	No	Yes
Decrease of wing length	NA	No	Laboratory water: yes Field-collected water: no	Yes
Microbial diversity in female adult^*d*^	Low	High	Laboratory water: low Field-collected water: high	Laboratory water: low Field-collected water: high

^*a*^ The specific results were indicated when outcomes differed between the waters.

^*b*^ Fish food (FF) is a diet commonly used in mass mosquito rearing.

^*c*^ Indicates a delay of more than 5 days for the complete achievement of each stage (until 100% of pupation or until 100% emergence) based on FF results (5 days needed to complete each stage).

^*d*^ Considered as “High” if there is no presence of a predominant genus representing more than 40% of the microbiota composition.

^*e*^ NA, not applicable.

^*f*^ FF used as reference to evaluate the difference between the diets for the different parameters.

^*g*^ RF, rabbit food.

All these diet-induced modifications should be carefully considered when designing research experiments to limit any associated bias or before implementing vector control activities that heavily rely on mosquito mass-rearing (i.e., sterile insect technique, *Wolbachia-*based population introgression strategy). For instance, diet influence on mosquito microbiota and size might bias the evaluation of virus transmission risk through vector competence changes (42, 45, 46). On the contrary, it could also influence *Ae. aegypti* flight ability or sexual competitiveness (29), phenotypic traits that are essential for alternative vector control strategies that require successful reproduction between released and wild-type mosquitoes (47). We, therefore, highlight the importance of optimizing rearing methods according to the expected outcomes to limit the bias introduced by mosquito laboratory rearing.

## MATERIALS AND METHODS

### Mosquitoes

The *Ae. aegypti* population was collected at the immature stage (larvae and pupae) in artificial larval sites in October 2021 in Petit-Bourg (16°13′04.9″N; 61°36′07.7″W), Guadeloupe. The immature stages (F_0_) were reared in dechlorinated tap water supplemented with brewer’s yeast capsules, and adults were fed *ad libitum* with 10% sucrose solution. Mosquitoes were reared under controlled conditions at 27 ± 1°C, 12/12 h light/dark photoperiod, and 70% relative humidity. To obtain the F_1_ generation used in this study, females were blood-fed twice a week using the Hemotek system (Hemotek, Ltd., UK).

### Diet description and nutrient quantification

Three diets commonly used in laboratory were selected: fish food (FF; TetraMin, Tetra, Germany), yeast (Gayelord Hauser, France), and rabbit food (RF; GMA, Guadeloupe). Manufacturers indicated that FF contains 46% protein, 11% fat, 3% cellulose, and 6% water, as well as several additives. For 4 g of yeast, the composition displayed by the manufacturer is the following: fat <0.5 g, protein 1.4 g, carbohydrates 0.8 g, 47 kcal of energy value, and several additives. RF contains 16.43% protein, 5.89% fat, 14.94% cellulose, and also several additives. As the heterogeneity of the information provided by the manufacturers may be associated with different nutrient dosage techniques, a standardized nutrient quantification was performed in our study. As larvae filter the nutrients in the water (48), in our study, we estimated the whole macronutrient amount (proteins, lipids, and carbohydrates) using diet-derived solutions. For that, each diet was ground, and a solution at 40g/L was prepared in 1 L of distilled water. The solutions were sent to the AgroQual laboratory that subcontracted the analysis to the Capinov laboratory. The Capinov laboratory is accredited by French authorities for food analysis, including nutrient composition quantification (ISO9001 and Cofrac n°1-6211). Nutrient quantification obtained by these laboratories is reported in Table 2.

**TABLE 2:** Nutrient composition of fish food, rabbit food, and yeast obtained by analysis of a solution at 40 g/L

Nutrient	Fish food	Rabbit food	Yeast
Protein (g/100 g)	1.6 (±0.4)	<0.50	1.0 (±0.4)
Lipid (g/100 g)	0.6 (±0.5)	<0.50	<0.50
Carbohydrate (g/100 g)	1.2	2.0	1.7
Caloric value (kcal/100 g)	16.7	8.0	10.7
Energy value (kcal/100 g)	70.0	33.8	45.6

### Experimental rearing conditions

Three commercial diets and one mix of two of them (mix 1:1 of FF and RF; mix FF/RF) were selected for this study (Fig. 1). Each diet was ground, and aliquots of 0.2 g were prepared and autoclaved before use in the experiments.

In total, eight rearing conditions were used in this study, including all combinations between two waters and four diets. Laboratory water collected in 2021 consisted of dechlorinated tap water (chlorinated tap water left standing for at least 72 h), while field-collected water was taken from two drums in November 2021 in Lauricisque (16°15′09.3″N; 61°32′53.6″W), Pointe-à-Pitre, Guadeloupe. Once in the laboratory, both field water samples were mixed (1:1) to obtain the field water used in this study. Water was stored at 27 ± 1°C before use.

The four diets were separately used to feed the larvae. Containers were disinfected with 70% ethanol before transferring 1 L of water (either laboratory dechlorinated tap water or field-collected water). F_1_ *Ae. aegypti* eggs were transferred in batches in the containers, and 0.2 g of diet was added to each container. Three days after the hatching, 250 first-instar larvae were counted and placed in a new container containing 1 L of water and 0.2 g of the same diet. Water and diet were renewed every 3 days.

For each condition (four diets and two rearing waters per diet), two biological replicates were performed. To reduce experiment bias and optimize the standardization of environmental conditions, rearing with the four diets was conducted in parallel for each water type. Larvae were reared under controlled conditions at 27 ± 1°C, 12/12 h light/dark photoperiod. Daily, the number of pupae and adults was counted until the end of the pupation or the end of the emergence. The pupation rate was estimated as the proportion of pupae counted per day among the total number of larvae initially placed in the container, while the emergence rate referred to the proportion of adults counted daily among the number of pupae previously counted.

Pupation and emergence curves were generated using nonlinear regression. An ordinary one-way ANOVA, followed by a Tukey’s test, was used to compare 50% pupation and 50% emergence values for each diet using both replicate data.

### Survival assays

Female survival was estimated for each of the eight diet/water combinations using between 21 and 30 females randomly collected from the rearing replicates. The emerging females were maintained under controlled conditions at 27 ± 1°C, 12/12 h light/dark photoperiod, and 70% relative humidity and fed *ad libitum* with 10% sucrose solution previously autoclaved. Deaths were recorded daily for 90 days. *Ae. aegypti* survival curves were generated by the Kaplan-Meier method and analyzed using a log-rank test and a Gehan-Breslow-Wilcoxon test.

### Wing measurement

To assess the influence of water/diet combinations on mosquito body size, wing measurements were conducted on mosquitoes. After adult emergence, 40 females and 20 males were randomly collected at equal proportions from both replicates for each diet/water combination. Wing dissection was performed using a scalpel and a binocular magnifying glass. Wing length considered as a proxy to evaluate mosquito size (49) was measured from the tip to the distal end of the allula as previously described by Dickson et al*.* (13) using ImageJ. To evaluate the influence of diet on *Ae. aegypti* wing length, an ordinary one-way ANOVA, followed by a Tukey’s test, was performed after verification of data normal distribution by Shapiro-Wilk test.

### Bacterial DNA extraction from water and mosquito samples

To identify the bacterial microbiota associated with the eight rearing conditions (four diets/two waters), 2 mL of water was collected before diet addition (*N* = 2) and from each container (*N* = 16) at the fourth instar larvae stage. Water was then centrifuged at 8,000 rpm for 10 min at 4°C. Supernatant was removed, and pellets were kept at −20°C before analysis. For each replicate, 10 fourth instar larvae and 10 females not previously fed with sugar or blood were randomly collected, separately pooled per sample type, and stored at −20°C, as for the rearing water. Before the DNA extraction, mosquitoes (larvae and adults) were surface-sterilized as previously described by Hery et al. (14). Briefly, whole mosquitoes were rinsed three times using 2 mL of sterile water, one time using 2 mL of 70% ethanol for 10 min, five times using sterile water, and finally one time using 0.8% NaCl. Then, a mechanical lysis was performed in 400 µL of sterile phosphate-buffered saline (PBS) using a bead beater (MM 400, Retsch, France) at 30 Hz for 30 s (three times) and a centrifugation at 12,000 pm for 3 min. Total genomic DNA was extracted from the water and the mosquitoes using the RNeasy PowerMicrobiome Kit following the manufacturer’s instructions (Qiagen, Germany). Extraction checking was performed using 16S rDNA universal bacteria primers 27F (5′ AGA GTT TGA TCC TGG 3′), 1492R (5′ GGT TAC CTT GTT ACG 3′) (50) and DreamTaq DNA polymerase (Thermo Scientific, USA) according to the manufacturer’s instructions. Amplicons were visualized under ultraviolet light by 1.5% agarose gel electrophoresis stained with gel red (Biotium, USA).

### Bacterial microbiota analysis

The 16S rRNA paired-end sequencing (300 bp read length) was performed using the universal prokaryote-specific primers targeting the V3–V4 region: 341F (5′CCT ACG GGN GGC WGC AG3′) and 785R (5′GAC TAC HVG GGT ATC TAA TCC3′) (51) with Illumina MiSeq at the Biomics Platform, C2RT (Institut Pasteur, Paris, France). After sequencing, the R package *DADA2* was used for cleaning raw sequences obtained (fastq) (52–54). This step includes filtering, merging, clustering, chimera and singleton deletion, as well as taxonomic ASV assignation. The taxonomic affiliations were deleted if the bootstrap value was lower than 50%. The Silva database was used for the taxonomy assignment (55). After the standardization, a total of 1,860 ASVs were obtained, and 122,343.5 reads per sample were retained for laboratory water (*N* = 26). For field water, 3,679 ASVs were obtained, and 88,158 reads per sample were maintained (*N* = 26). Metrics as the alpha (Chao1, Shannon, and Simpson) and the beta (Bray-Curtis dissimilarity matrix; PCOA representation) diversity metrics were generated using R packages *ggplot2* (56), *phyloseq* (57), and *vegan* (58). For alpha and beta metrics, statistical analysis was performed using ANOVA, a post-hoc Tukey test, and PERMANOVA.

### Statistical analysis

The level of statistical significance for all analyses on *Ae. aegypti* life traits was set at *P* ≤ 0.05. Graphs and statistical analyses were carried out in GraphPad Prism v9 (GraphPad Software Inc.) and R version 4.3.0 (R Core Team, Vienna, Austria).

## Data Availability

The raw sequencing data from the 16S rRNA gene metabarcoding analysis presented in the study are available from NCBI under BioProject accession number PRJNA1345384.
